# Temporomandibular joint disorders, happiness, and COMT, HTR2A and FKBP5 polymorphisms in adolescents: a cross-sectional study

**DOI:** 10.1590/1807-3107bor-2025.vol39.032

**Published:** 2025-05-23

**Authors:** Ana Luiza Peres BALDIOTTI, Gabrielle AMARAL-FREITAS, Mariane Carolina Faria BARBOSA, Paula Rocha MOREIRA, Renata de Castro MARTINS, Renato Assis MACHADO, Ricardo Della COLETTA, Michelle Nascimento MEGER, Saul Martins PAIVA, Rafaela SCARIOT, Fernanda de Morais FERREIRA

**Affiliations:** (a) Universidade Federal de Minas Gerais – UFMG, School of Dentistry, Department of Pediatric Dentistry, Belo Horizonte, MG, Brazil.; (b) Universidade Federal de Minas Gerais – UFMG, Institute of Biological Sciences, Department of Morphology, Belo Horizonte, MG, Brazil.; (c) Universidade Federal de Minas Gerais – UFMG, School of Dentistry, Department of Social and Preventive Dentistry, Belo Horizonte, MG, Brazil.; (d) Universidade Estadual de Campinas – Unicamp, School of Dentistry of Piracicaba, Department of Oral Diagnosis, Piracicaba, SP, Brazil.; (e) Universidade Positivo, School of Health and Biological Sciences, Curitiba, PR, Brazil.; (f) Universidade Federal do Paraná – UFPR, School of Dentistry, Department of Stomatology, Curitiba, PR, Brazil.

**Keywords:** Temporomandibular Joint Disorders, Happiness, Polymorphism, Genetic, Adolescent

## Abstract

Temporomandibular joint disorder (TMD) has a multifactorial etiology involving psychological and genetic aspects. This condition commonly begins in adolescence, which is a period of emotional, physical, and psychological maturation. The aim of the present study was to investigate associations between temporomandibular joint (TMJ) disorder and happiness as well as polymorphisms in the *COMT*, *HTR2A*, and *FKBP5* genes in Brazilian adolescents. A cross-sectional study was conducted with 90 adolescents aged 13 to 18 years. TMD was diagnosed using the RDC/TMD. The Subjective Happiness Scale (SHS) was used to assess happiness. Saliva samples were collected for the analysis of genomic DNA and genotyping of single nucleotide polymorphisms in the *COMT* (rs165656, rs174675), *HTR2A* (rs6313, rs4941573), and *FKBP5* (rs1360780, rs3800373) genes using real-time PCR (Taqman method). Bivariate, unadjusted, and adjusted binary logistic regression analyses were performed (p < 0.05). Happiness was associated with TMD in the adolescents (OR=1.37; 95%CI: 1.02–1.85; p = 0.037). The rs174675 polymorphism in the *COMT* gene was significantly associated with TMD (OR = 0.18; 95%CI: 0.04–0.74; p = 0.018). No associations were found between TMD and polymorphisms in *HTR2A* and *FKBP5* genes (p > 0.05). TMD was associated with happiness, as adolescents who considered themselves less happy were more likely to have this disorder. The diagnosis of TMD was also associated with the rs174675 polymorphism in the *COMT* gene, as the prevalence of the disorder was higher among homozygous C Brazilian adolescents than in heterozygous CT individuals.

## Introduction

Temporomandibular joint disorder (TMD) describes a set of skeletal and neuromuscular conditions in the craniofacial region^1,2^and is the most common cause of non-dental orofacial pain.^2^ It may have different diagnoses classified as muscular disorders (myofascial pain or myofascial pain with limited mouth opening), disc displacements (disc displacement which may be with or without reduction), and joint disorders (arthralgia, osteoarthritis, or osteoarthrosis).^1,3^

The etiology of TMD is complex. The multifactorial biopsychosocial model of TMD is currently the most widely accepted etiology, in which biological, psychological, and social factors act together.^3,4^ Emotional factors, such as stress, anxiety, and depression, play an important role in the origin and evolution of symptoms.^2,4,5^ Cohort studies involving adults and adolescents have demonstrated that depression, perceived stress, bad mood, somatization, and dissatisfaction with life increase the risk of TMD and the initiation or perpetuation of pain related to this disorder.^6,7^

TMD can begin in childhood and adolescence.^8,9^ We hypothesize that the onset of TMD is influenced by the characteristics of adolescence, which is a period of emotional, pubertal, psychological, and social maturation when individuals are more vulnerable to the emergence of psychopathologies due to increased emotional intensity and immature cognitive control.^8,9^

The dynamics of TMD are modulated by genetic factors, with gene-environment interactions involved in the emergence of the disorder.^4,10,11^ Genetic association studies have investigated the role of polymorphisms in the etiology of TMD. The findings indicate several candidate genes that have small effects and determine the course and outcome of the disorder.^10,11,12^ While such studies have interesting results, the exact genes and polymorphisms associated with risk of or protection from TMD remain unknown.^4^ Genes involved in the regulation of the serotonergic system have attracted attention due to their role in nociceptive and affective pathways.^12^Polymorphisms in the catechol-O-methyltransferase (*COMT*), 5-hydroxytryptamine receptor 2A (*HTR2A*), and FKBP prolyl isomerase 5 (*FKBP5*) genes have a potential association with this system.^1,4,12,13^

The *COMT* gene encodes the catechol-O-methyltransferase enzyme, which catalyzes the degradation and reuptake of a wide range of catechols, including catecholamines (dopamine, adrenaline, and noradrenaline).^4,14,15^ Polymorphisms in this gene are associated with emotional disorders,^16^ anxiety,^17^ and the pathophysiology of TMD.^4,12,17^ The *HTR2A* gene encodes the serotonin receptor 5-hydroxytryptamine, which is an important central nervous system neurotransmitter that regulates various physiological and cognitive functions.^18,19^ Its variants have been associated with TMD.^12,18^ bruxism.^20^ obstructive sleep apnea,^21^ and psychiatric disorders.^22^ The FKBP prolyl isomerase 5 (*FKBP5*) gene plays a role in the sensitivity of the glucocorticoid receptor and therefore regulates the stress response system.^23,24^ *FKBP5* polymorphisms are associated with risk of developing post-traumatic stress disorder,^25^ depression,^23,24^ anxiety,^23^ and greater surgical discomfort during third molar extractions.^24^

Based on the notion that adolescence influences susceptibility to the emergence of emotional disorders and impacts happiness^8,9^ and because the first signs and symptoms of TMD often appear in childhood and adolescence, the aim of the present study was to investigate associations between temporomandibular joint disorder (TMJ) happiness, and polymorphisms in the *COMT* (rs165656, rs174675), *HTR2A* (rs6313, rs4941573), and *FKBP5* (rs1360780, rs3800373) genes in adolescents. Studies involving this period of life can contribute to a better understanding of the onset and evolution of TMD.^26,27^ To date, however, few have investigated the role of these polymorphisms in the etiology of TMJ among adolescents.^4^

## Methods

### Ethics approval

This study was conducted in accordance with the STREGA statement (https://www.equator-network.org/reporting-guidelines/strobe-strega/) and ethical precepts laid down in the Declaration of Helsinki. The study was approved by the Human Research Ethics Committee of the Federal University of Minas Gerais (Protocol #01936918.8.0000.5149). Parents/caregivers or adolescents aged 18 years received written information on the study and signed as statement of informed consent. Adolescents under 18 years of age signed a statement of informed acceptance.

### Study population

A cross-sectional study was conducted with a sample of 90 Brazilian adolescents aged 13 to 18 years. Biologically unrelated male and female individuals with or without TMJ were included. Adolescents undergoing orthodontic treatment and those with syndromic or cognitive disorders were excluded.

### Data collection

Two examiners (A.L.P.B. and G.A.F.) who had undergone training and calibration performed the clinical examinations and administered the instruments. Training and calibration were conducted by researchers experienced in the use of the clinical indexes employed in this study. The examiners received theoretical and practical training involving clinical examination of 28 patients. Inter-examiner and intra-examiner agreement (kappa coefficient) for the diagnosis of TMJ was 0.907 and 0.804, respectively.

Prior to the main study, a pilot study was conducted with 10 adolescents to test the proposed methods. Based on the pilot study, the methods were maintained and the individuals who participated in this stage were included in the main study.

Data collection took place between May and December 2019. The participants were recruited at the dental clinic of the Federal University of Minas Gerais.

A self-administered questionnaire was developed for this research based on items used in similar studies. There were questions about the adolescents’ socioeconomic and demographic data (name, age, birthday, sex) and oral health-related data (previous dental trauma, sleep and awake bruxism). Then the adolescents were examined in a dental chair under artificial light by an examiner wearing personal protective equipment and using a sterilized clinical kit consisting of a mouth mirror and WHO dental probe. The examiners looked for signs of tooth wear and dental trauma. Positive signs of tooth wear and a parental report of teeth grinding and clenching during the day or night were considered indicative of probable awake or sleep bruxism, respectively. A questionnaire addressing socioeconomic, demographic, and health-related characteristics was administered to the adolescents.

The diagnosis of TMJ was performed using Axis I of the validated Brazilian version of the Research Diagnostic Criteria for Temporomandibular Disorders (RDC/TMD) to detect muscle disorder, disc displacement, and/or joint disorder^29^. We focused on joint disorders in the present study. Thus, adolescents with arthralgia, osteoarthritis, and osteoarthrosis (separately or concomitantly) were considered to have TMD. We did not use the Diagnostic Criteria for Temporomandibular Disorders (DC/TMD) tool because it had not yet been validated in Brazilian Portuguese at the time of data collection.

The participants also answered the Brazilian version of the Subjective Happiness Scale (SHS),^29,30^ which is a self-report measure of subjective global happiness. The four-item SHS assesses whether the respondents consider themselves happy or unhappy based the number marked on a seven-point scale indicating the extent to which each of the four statements applies to them. The scale increases from one to seven. A higher score on the first three items indicates a greater degree of happiness, whereas a higher score on the last item indicates a lower degree of unhappiness.^29,30^

### DNA samples and genotyping

DNA was analyzed for genotyping. The adolescents rinsed their mouths with 5 mL of a 3% glucose solution for one minute and then spat the liquid into Falcon tubes to obtain cells from the oral epithelium. 15-mL centrifuge tubes (Corning Inc, Corning, USA) were filled with expectorated saliva samples. The protocol established by Kuchler et al.^31^ was followed for the extraction of DNA.

The selection of polymorphisms in genes was performed based on previous candidate-gene identification studies^10,12,18,24^ for the pathophysiology of TMD. Gene characterization and polymorphisms are described in Table 1. Polymorphisms in the *COMT* (rs165656, rs174675), *HTR2A* (rs6313, rs4941573), and *FKBP5* (rs1360780, rs3800373) genes were determined using polymerase chain reaction (PCR) analysis, employing TaqMan method in a real-time PCR system (Applied Byosistems^®^, 7500 Real-Time PCR System, Thermo Fisher Scientiﬁc, Foster City, USA).

**Table 1:** Studied polymorphisms and their characteristics.

Gene	Polymorphisms	Position	MAF	Alteration
COMT	rs165656	22q11.21	0.465	G>A/C/T
	rs174675	22q11.21	0.339	T>C
HTR2A	rs6313	13q14.2	0.403	G>A/C
	rs4941573	13q14.2	0.364	A>C/G
FKBP5	rs1360780	6p21.31	0.340	T>A/C
	rs3800373	6p21.31	0.334	C>A/G

Obtained from: http://www.ncbi.nih.gov. MAF: Minor Allele Frequency.

### Statistical analysis

The characterization of the sample was performed using descriptive statistics. Unadjusted and adjusted binary logistic regression models were run between the outcome (TMJ) and variables of interest considering the underlying theoretical framework. Variables with a p-value < 0.2 in the bivariate analysis were incorporated into the multivariate model. Variables were also incorporated taking into account the theoretical framework. Wald’s backward method was used to create the final model, generating adjusted odds ratios (OR) and respective 95% confidence intervals (CI) for TMJ according to categories of the independent variables. A p-value < 0.05 was considered indicative of statistical significance. Data analysis was performed with the Microsoft Excel and Statistical Package for the Social Sciences (SPSS, version 22.0, IBM Corp., Armonk, USA).

## Results

### Descriptive statistics

One hundred and five adolescents were recruited for the study, 90 of whom returned the signed informed consent, agreed to participate, and underwent all phases of the study (response rate: 85.7%) (Figure). Forty-six (51.1%) participants were girls and 44 (48.9%) were boys. Mean age was 15.9 years, 73% were non-white and 27% were white, 36% of parents/caregivers had studied 8 years or less, 30.5% of the adolescents’ families received up to 1 Brazilian minimum wage per month (exchange rate was 1 to US$ 196,84 at the time of data collection), and 56.1% received 1 to 3 Brazilian minimum wages per month. The prevalence of TMJ was 41.1% (n = 37). Twenty-three participants with symptoms of TMJ (62.2%) were girls and 14 (37.8%) were boys. Table 2 displays the frequency of each variable.

**Figure:**
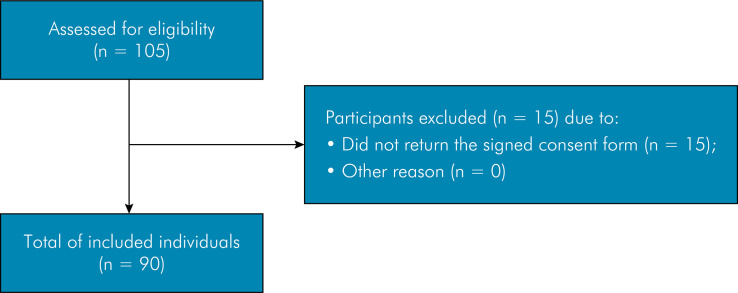
Flowchart of study stages.

**Table 2:** Prevalence and confidence interval of the measured variables.

Predictive variables	Percentiles		
	P25	P50	P75
I consider myself happy (Feeling of happiness - question one)	4.00	6.00	7.00
I consider myself less happy than my friends (Feeling of happiness - question two)	4.00	5.50	6.00
I don’t consider myself happy in general (Feeling of happiness -question three)	4.00	4.50	6.00
I’m never as happy as I could be (Feeling of happiness - question four)	2.00	4.00	4.00
Predictive variables	Prevalence (%)		
	(95%CI)		
*COMT* (rs174675)			
CC	69 (77.5)		
CT	20 (22.5)		
TT	0		
*COMT* (rs165656)			
GG	24 (26.5)		
CG	44 (49.4)		
CC	21 (23.6)		
*HTR2A* (rs6313)			
AA	17 (18.9)		
AG	50 (55.6)		
GG	22 (25.5)		
*HTR2A (rs4941573)*			
*AA*	27 (30.3)		
*AG*	45 (50.6)		
*GG*	17 (19.1)		
*FKBP5 (rs1360780)*			
*CC*	40 (44.9)		
*CT*	38 (42.7)		
*TT*	11 (12.4)		
*FKBP5* (rs3800373)			
AA	43 (47.8)		
AC	36 (40.4)		
CC	10 (11.2)		
Dental trauma			
Yes	8 (9.6)		
No	75 (90.4)		
Probable awake bruxism			
Yes	6 (6.8)		
No	82 (93.2)		
Probable sleep bruxism			
Yes	11 (12.5)		
No	77 (87.5)		

*Categories that do not add up to N=90 had missing data.

### Analytical statistics

In the binary logistic regression, the fourth item on the Subjective Happiness Scale was associated with TMJ (p = 0.022). For every point higher on the SHS, the risk of TMJ increases by 36%. The rs174675 recessive C *COMT* gene polymorphism (p = 0.011) and probable sleep bruxism (p = 0.038) were also associated with TMJ in Brazilian adolescents in the binary logistic regression (Table 3).

**Table 3:** Binary logistic regression of TMJ and the measured variables.

Variables	Temporomandibular Joint Disorders						OR	95%CI	p-value
	Present			Absent					
	P 25	P 50	P 75	P 25	P 50	P 75			
I consider myself happy (Feeling of happiness - question one)	4.00	5.00	7.00	4.00	6.00	7.00	0.97	0.77-1.24	0.837
I consider myself less happy than my friends (Feeling of happiness - question two)	4.00	4.00	6.00	4.00	6.00	7.00	0.76	0.58-1.00	0.054
I don’t consider myself happy in general (Feeling of happiness - question three)	3.00	4.00	6.00	4.00	5.00	6.00	0.84	0.64-1.08	0.178
I’m never as happy as I could be (Feeling of happiness - question four)	2.00	4.00	5.00	1.00	3.00	4.00	1.36	1.04-1.78	**0.022**
	n (%)			n (%)					
Sex							2.14	0.91-5.05	0.082
Female	23 (62.2)			23 (43.4)					
Male (ref)	14 (37.8)			30 (56.6)					
*COMT* (rs174675)									
CT (ref)	3 (8.1)			17 (32.7)			5.50	1.48-20.50	**0.011**
CC	34 (91.9)			35 (67.3)					
TT	0			0					
*COMT* (rs165656)									
GG (ref)	9 (24.3)			15 (28.8)					
CG	17 (45.9)			27 (51.9)			1.05	0.38-2.93	0.927
CC	11 (29.7)			10 (19.2)			1.83	0.56-6.03	0.318
*HTR2A* (rs6313)									
AA (ref)	8 (21.6)			9 (17.3)					
AG	20 (54.1)			30 (57.7)			0.75	0.25-2.27	0.611
GG	9 (24.3)			13 (25.0)			0.78	0.22-2.79	0.701
*HTR2A* (rs4941573)									
*AA* (ref)	9 (24.3)			18 (34.6)					
*AG*	20 (54.1)			25 (48.1)			1.60	0.59-4.32	0.354
*GG*	8 (21.6)			9 (17.3)			1.78	0.51-6.17	0.365
*FKBP5* (rs1360780)									
*CC* (ref)	16 (40.0)			24 (60.0)					
*CT*	17 (44.7)			21 (55.3)			1.21	0.49-2.98	0.672
*TT*	4 (36.4)			7 (63.6)			0.86	0.21-3.41	0.827
*FKBP5* (rs3800373)									
AA (ref)	19 (51.4)			24 (46.2)					
AC	14 (37.8)			22 (42.3)			0.80	0.33-1.98	0.635
CC	4(10.8)			6 (11.5)			0.84	0.20-3.42	0.810
Dental trauma							2.80	0.62-12.61	0.181
Yes (ref)	5 (15.2)			3 (6.0)					
No	28 (84.8)			47 (94.0)					
Probable awake bruxism							8.23	0.92-73.72	0.060
Yes	31 (86.1)			51 (98.1)					
No (ref)	5 (13.9)			1 (1.9)					
Probable sleep bruxism							4.41	1.08-17.98	**0.038**
Yes	8 (21.6)			3 (5.9)					

*Categories that do not add up to N=90 had missing data.

After adjusting for other variables in the multiple regression analysis (Table 4), TMJ was associated with the rs174675 recessive C *COMT* polymorphism (p = 0.018), indicating that people without the rs174675 recessive C *COMT* polymorphism had 82% less chance of presenting joint disorder. Also, the feeling of never being as happy as the they could be (fourth item on the SHS) was associated with joint disorder (p = 0.037), so for each point higher on the SHS, the risk of TMJ increased by 37%.

**Table 4:** Multiple logistic regression models for TMJ in adolescents.

Predictive variables	Adjusted OR	95%CI	p-value
Sex			
Male (ref)			
Female	1.64	0.61-4.40	0.327
I consider myself happy (Scale 1 to 7)	1.10	0.78-1.56	0.572
I consider myself less happy than my friends (Scale 1 to 7)	0.84	0.61-1.17	0.311
I don’t consider myself happy in general (Scale 1 to 7)	0.92	0.66-1.30	0.649
I’m never as happy as I could be (Scale 1 to 7)	1.37	1.02-1.85	**0.037**
*COMT* (rs174675)			
CC (ref)			
CT/TT	0.18	0.04-0.74	**0.018**

Bold values indicate p-values < 0.05.

## Discussion

The etiology of TMD is multifactorial, involving physical, psychological,^5^ and genetic factors.^4,12^ The present study evaluated associations between TMJ, feelings of happiness, as well as polymorphisms in the *COMT*, *HTR2A*, and *FKBP5* genes in Brazilian adolescents. As TMD is a complex condition with many diagnoses and different etiologies, we chose to study one group (TMJ) in order to be more specific and accurate about the possible associated aspects. As far as we know, this is the first study to evaluate the association between these factors and TMJ. The role of psychological and genetic factors in the etiology of TMJ requires further clarification.^4,12^ We hypothesized that emotional aspects and polymorphisms in the *COMT*, *HTR2A*, and *FKBP5* genes contribute to the etiology of TMJ, as these factors can affect functions that may influence the condition.

Adolescence is a time of numerous changes.^8^ Thus, the development of mental problems, such as anxiety, unhappiness, and depression, is not uncommon in this period.^9^ A recent study found an association between TMD and depression,^32^ which draws attention to the influence of emotional factors. Another study showed that oral conditions can decrease the level of happiness in Brazilian adolescents.^33^ Happiness is a construct similar to the subjective feeling of well-being and is considered an important factor in psychology as a complementary construct of health mental. Thus, the investigation of feelings of happiness constitutes a subjective assessment of whether a person is happy or unhappy.^29^ In the present study, we found a significant association between happiness and TMJ, as adolescents who reported never being as happy as they could be (fourth item on Subjective Happiness Scale) were more likely to have TMJ. To the best of our knowledge, this is the first study to assess this issue and it may contribute to future discussions regarding the impact of subjective feelings of happiness on the etiology of TMJ and the emergence of pain related to this disorder.

The main findings of this study were the associations of TMJ with feelings of happiness and the associations of TMJ and polymorphisms in the *COMT* gene in adolescents. This gene is an important candidate for the etiology of painful conditions,^17^ as it regulates the expression of the enzyme catechol-O-methyltransferase, which is responsible for metabolizing the active catecholamine derivative and promoting the inactivation of acetylcholine.^15,15^Polymorphisms in the *COMT* gene are responsible for the malfunction of this enzyme, resulting in low reuptake and increased levels of catecholamines^34,35^ as well as the stimulation of β adrenergic receptors responsible for pain sensitivity.^36^

A recent systematic review with meta-analysis found that polymorphisms in the *COMT* gene may be associated with TMD.^4^ In the present study, we found that the rs174675 polymorphism in the *COMT* gene was associated with TMJ, as the prevalence of the disorder was higher among adolescents with this polymorphism. As far as we know, this polymorphism has not previously been investigated for its association with TMJ and may be included with other candidate polymorphisms associated with this condition.^4,12^

Polymorphisms in the *HTR2A* gene were also investigated in the present study. This gene encodes the serotonin receptor 5-hydroxytryptamine, which is considered an essential component of the serotonergic system, as it involves physiological functions, such as memory, sleep, nociception, feeding, and reward.^18,19^ The modulation of the functioning and activity of *HTR2A* is poorly understood, but this gene is believed to play a pro-nociceptive role in spinal pain by modulating serotonergic activity, causing an increase in nociceptive transmission that results in hyperalgesia and increased levels of continuous (neuropathic) pain.^37^ As TMD is the main cause of chronic non-dental orofacial pain,^2^ we hypothesized that it is associated with polymorphisms in *HTR2A*. Freitas et al.^18^ evaluated this association in Brazilian adults and found that the 102T-C polymorphism was associated with TMD.^18^ We found no studies that evaluated *HTR2A* polymorphisms in Brazilian adolescents, which demonstrates the importance of the present investigation. However, polymorphisms in the *HTR2A* gene were not associated with TMJ in the adolescents in our study.

To the best of our knowledge, this is the first study to evaluate the association between polymorphisms in the *FKBP5* gene and TMJ. We found only two previous studies that investigated the association of such polymorphisms with oral conditions. Reis et al.^24^found an association between the rs3800373 polymorphism in the *FKBP5* gene and greater surgical discomfort associated with third molar surgery in women. Scariot et al.^34^ investigated the association between polymorphisms (rs1360780, rs3800373) in the *FKBP5* gene and bruxism in children. We speculated that polymorphisms in the *FKBP5* gene would be associated with TMJ in adolescents due to the emotional and psychological aspects that influence the etiology of TMD^4,5^ and the fact that polymorphisms in this gene have previously been associated with post-traumatic stress disorder,^25^ anxiety,^23^ depressive disorder,^23,24^ and surgical discomfort.^24^ However, TMJ was not associated with polymorphisms in the *FKBP5* gene in the present study.

In this study, we did not evaluate the direct association between feelings of happiness and polymorphisms in the *COMT*, *HTR2A*, and *FKBP5* genes. However, future studies should assess the connection between these factors since these genes are linked to regulatory functions in the central nervous system, such as responses to stress, emotional aspects such as anxiety and depression, and subjective aspects such as quality of life and oral health-related quality of life.^24,40-45^ Furthermore, future studies should include a larger sample of adolescents, with the measurement of more comprehensive emotional aspects, and the evaluation of other polymorphisms in the same genes and/or different candidate genes to better understand the etiology of TMJ in this population.

This study provides useful information on emotional and genetic aspects in the development of TMJ in adolescents, which is important, considering the limited research on this condition in this specific age group. Moreover, as gene expression depends on life experiences,^17^ gene-environment interactions, especially with regards to stress and psychological problems, are likely less common among younger individuals in comparison to adults and older people. We used international criteria for the diagnosis of TMD^28,38,39^ and the assessment of feelings of happiness.^30,31^ We also had excellent kappa agreement coefficients, which indicates the reliability of the data. However, caution should be used when interpreting the results, as the study was conducted with a convenience sample and we only measured one subgroup (TMJ) of the three main TMD types (myofascial pain, disc displacement, and TMJ). Moreover, psychological and emotional aspects of the adolescents that could influence their perception of happiness were not investigated.

## Conclusion

The present study found an association between TMJ and happiness in Brazilian adolescents, as adolescents who considered themselves less happy were more likely to have TMJ compared to those who considered themselves happier. The diagnosis of TMJ was also associated with a polymorphism in the *COMT* gene (rs174675), as homozygous C participants were more likely to have TMJ than heterozygous CT individuals.
